# Supplementary material for: Effect of blood-flow restricted vs heavy-load resistance training on strength, power, and speed for healthy volunteers: a systematic review and meta-analysis
Source: PeerJ. 2025 Mar 18;13:e19110. doi: 10.7717/peerj.19110 (PMC11927561; doi:10.7717/peerj.19110)
Supplement: Supplemental Information 1 [file peerj-13-19110-s001.docx]

**Search Alert**

Pubmed (460)

("blood flow restriction"[Title/Abstract] OR "vascular occlusion"[Title/Abstract] OR "KAATSU"[Title/Abstract]) AND ("strength training"[Title/Abstract] OR "resistance training"[Title/Abstract] OR "high intensity training"[Title/Abstract] OR "weight training"[Title/Abstract] OR "high load training"[Title/Abstract])

SCOPUS (918)

[Article title, Abstract, Keywords] ("blood flow restriction" OR "vascular occlusion" OR "KAATSU"); AND [Article title, Abstract, Keywords] ("strength training" OR "resistance training" OR "high intensity training" OR "weight training" OR "high load training")

SPORTDiscus (277)

Search Alert: ("AB ("blood flow restriction" OR "vascular occlusion" OR "KAATSU") AND AB ("strength training" OR "resistance training" OR "high intensity training" OR "weight training" OR "high load training")

Web of Science (1092)

[Topic] ("blood flow restriction" OR "vascular occlusion" OR "KAATSU") AND [Topic] ("strength training" OR "resistance training" OR "high intensity training" OR "weight training" OR "high load training")

CNKI (178)

[Topic] ("blood flow restriction" OR "vascular occlusion" OR "KAATSU") AND [Topic] ("strength training" OR "resistance training" OR "high intensity training" OR "weight training" OR "high load training")
