# Supplementary material for: Effect of blood-flow restricted vs heavy-load resistance training on strength, power, and speed for healthy volunteers: a systematic review and meta-analysis
Source: PeerJ. 2025 Mar 18;13:e19110. doi: 10.7717/peerj.19110 (PMC11927561; doi:10.7717/peerj.19110)
Supplement: Supplemental Information 3 [file peerj-13-19110-s003.docx]

**Means and Standard Deviations (data)**

| **Study** | **Year** | **Outcomes (pre and post)** | **Outcomes (change)** |
| --- | --- | --- | --- |
| Bradley | 2023 | 1RM deadlift: BFR, 41.1 ± 14.4, 49.3 ± 17.4; HLR, 35.2 ± 12.4, 41.7 ± 14.6 Maximum power: BFR, 222.2 ± 72.3, 250 ± 66.1; HLR, 209.1 ± 74.4, 229.6 ± 66.9 | 1RM deadlift: BFR, 8.2 ± 16.1; HLR, 6.5 ± 13.6 Maximum power: BFR, 27.8 ± 69.5; HLR, 20.5 ± 70.9 |
| Cattilla-Lopez | 2023 | CMJ: BFR, 38.8 ± 3.8, 40.9 ± 4.8; HLR, 37.2 ± 3.8, 39.2 ± 5.8 MVC vertical force: 1998.3 ± 262, 2088.3 ± 264.2; HLR, 2003 ± 144.8, 2012.4 ± 154.1 peak power: 24.9 ± 2.3, 24.3 ± 3; HLR, 30.3 ± 8, 26.7 ± 3.5 30-m sprint: 4.4 ± 0.2, 4.3 ± 0.1; HLR, 4.5 ± 0.1, 4.3 ± 0.1 | CMJ: BFR, 2.1 ± 4.4; HLR, 2 ± 5.1 MVC vertical force: 90 ± 263; HLR, 9.4 ± 149.7 peak power: -0.6 ± 2.7; HLR, -3.6 ± 6.9 30-m sprint: -0.1 ± 0.2; HLR, -0.2 ± 0.2 |
| Centner | 2019 | MVC (plantar flexors): BFR, 226.7 ± 47.6, 248.9 ± 48.5; HLR, 189.0 ± 83.1,214.6 ± 86.1 | MVC (plantar flexors): BFR, 22.2 ± 4.8; HLR, 25.6 ± 6.3 |
| Centner | 2023 | MVC (plantar flexors): BFR, 114.8 ± 30.0, 164.7 ± 48.8; HLR, 121.8 ± 40.7, 174.9 ± 34.8 | MVC (plantar flexors): BFR, 49.9 ± 42.6; HLR, 53.1 ± 38.1 |
| Clark | 2011 | Maximum Voluntary Contraction Force: BFR, 1103.4 ± 104.1, 1173.8 ± 90.8; HLR, 1273.5 ± 125.1, 1424.6 ± 130.7 | Maximum Voluntary Contraction Force: BFR, 70.4 ± 95.7; HLR, 151.1 ± 127.9 |
| Cook | 2018 | 1RM knee extension: BFR, 116 ± 21, 131 ± 24; HLR, 88 ± 32, 118 ± 44; | 1RM knee extension: BFR, 15 ± 22.7; HLR, 30 ± 40.1 |
| Cook | 2019 | MVC (leg extension): BFR, 115.9 ± 38.5, 128 ± 45.4; HLR, 103.8 ± 36.1, 126 ± 42.5 MVC (leg flexion): BFR, 63.4 ± 19.6, 69.5 ± 19.3; HLR, 60.7 ± 18.4, 68.9 ± 23 | MVC (leg extension): BFR, 12.1 ± 42.3; HLR, 22.2 ± 39.7 MVC (leg flexion): BFR, 6.1 ± 19.4; HLR, 8.2 ± 21.8 |
| Davids | 2021 | 1RM (leg extension): BFR, 19 ± 4.5; HLR, 19 ± 12.6；CMJ: BFR, 1.4 ± 2.1; HLR, 1.7 ± 2.4；CMJ power: BFR, 31 ± 189.1; HLR, 68 ± 158; SJ: BFR, -0.1 ± 2.6; HLR, 2.3 ± 2.6; SJ power: BFR, 21 ± 221.1; HLR, 101 ± 212.5 | 1RM (leg extension): BFR, 19 ± 4.5; HLR, 19 ± 12.6；CMJ: BFR, 1.4 ± 2.1; HLR, 1.7 ± 2.4；CMJ power: BFR, 31 ± 189.1; HLR, 68 ± 158; SJ: BFR, -0.1 ± 2.6; HLR, 2.3 ± 2.6; SJ power: BFR, 21 ± 221.1; HLR, 101 ± 212.5 |
| Early | 2020 | 1RM leg curl: BFR, 105 ± 20, 130 ± 26; HLR, 106 ± 26, 140 ± 32 1RM leg extension: BFR, 159 ± 34, 191 ± 39; HLR, 163 ± 44, 197 ± 55 1RM heel raise: BFR, 177 ± 64, 229 ± 64; HLR, 174 ± 55, 226 ± 74 | 1RM leg curl: BFR, 25 ± 23.6; HLR, 34 ± 29.5 1RM leg extension: BFR, 32 ± 36.8; HLR, 32 ± 50.4 1RM heel raise: BFR, 52 ± 50.4; HLR, 52 ± 67.3 |
| Guang | 2021 | 1RM Parallel Back Squat: BFR, 126.3 ± 17.1, 138.8 ± 18.1; HLR, 123.1 ± 13.3, 136.9 ±13.9 CMJ: BFR, 64.4 ± 2.3, 68.6 ± 2.3; HLR, 63.6 ± 2.7, 66.7 ± 2.7 Sprint: BFR, 3.62 ± 0.06, 3.50 ± 0.05; HLR, 3.64 ± 0.07 ± 2.7, 3.56 ± 0.06 | 1RM Parallel Back Squat: BFR, 12.5 ± 17.6; HLR, 13.8 ±13.6 CMJ: BFR, 4.2 ± 2.3; HLR, 4.1 ± 2.7 Sprint: BFR, -0.12 ± 0.06; HLR, -0.08 ± 0.07 |
| Horiuchi | 2023 | 1RM (Knee extension): BFR, 60.4 ± 6.6, 64.4 ± 6.0; HLR, 62.0 ± 6.4, 71.1 ± 6.5; 1RM (Leg press): BFR, 124 ± 16.7, 139 ± 13.9; HLR, 129 ± 14.1, 147 ± 13.5 | 1RM (Knee extension): BFR, 4 ± 6.3; HLR, 9.1 ± 6.5; 1RM (Leg press): BFR, 15 ± 15.5, HLR, 18 ± 13. |
| Jeon | 2022 | 1RM knee extension: BFR, 106.6 ± 25.4, 113.6 ± 22.8; HLR, 81.5 ± 20.7, 92.5 ± 21.6 1RM knee flexion: BFR, 43.4 ± 14.2, 53.3 ± 10.1; HLR, 33.7 ± 8.2, 42.0 ± 10.5 | 1RM knee extension: BFR, 7 ± 24.2; HLR, 11 ± 21.2 1RM knee flexion: BFR, 9.9 ± 12.7; HLR, 8.3 ± 9.6 |
| Jones | 2022 | vertical power: BFR, 32.8 ± 2.2, 33.3 ± 2.2; HLR, 33 ± 2.3, 33.8 ± 2.4 Hamstrings maximum force: BFR, 29.1 ± 42.3; HLR, 38.6 ± 39.9 | vertical power: BFR, 0.5 ± 2.2; HLR, 0.8 ± 2.4 Hamstrings maximum force: BFR, 29.1 ± 42.3; HLR, 38.6 ± 39.10 |
| Karabulut | 2010 | 1RM leg extension: BFR, 82.4 ± 4.4, 97.3 ± 4.1; HLR, 70.8 ± 4.3, 92.6 ± 5.5 1RM leg extension: BFR, 144.6 ± 6.4, 171.6 ± 6.2; HLR, 119.3 ± 5.9, 141.3 ± 7.5 | 1RM leg extension: BFR, 14.9 ± 4.3; HLR, 21.8 ± 5 1RM leg flexion: BFR, 27 ± 6.3; HLR, 22 ± 6.9 |
| Kim | 2009 | 1RM (leg press): BFR, 197.8 ± 13.7, 219.8 ± 22.1; HLR,195.3 ± 16.9, 224.5 ± 20.8; 1RM (knee flexion): BFR, 90.1 ± 7.1, 93.3 ± 7.9; HLR, 82.9 ± 5.2, 93.2 ± 6.4; 1RM (knee extension): BFR, 96.9 ± 7.9 , 103.8 ± 8.2; HLR, 93.5 ± 6.1, 111.0 ± 7.6 | 1RM (leg press): BFR, 22 ± 19.3, HLR, 29.2 ± 19.2; 1RM (knee flexion): BFR, 3.2 ± 7.5, HLR, 10.3 ± 5.9; 1RM (knee extension): BFR, 6.9 ± 8.1, HLR, 17.5 ± 7.0 |
| Kriley | 2014 | 1RM squat: BFR, 174.4 ± 22.2, 180.4 ± 24.8; HLR, 196.9 ± 35.1, 210.6 ± 38.9 Vertical jump: BFR, 80 ± 7.7, 81.5 ± 7.2; HLR, 70.6 ± 11.9, 73.7 ± 13.2 | 1RM squat: BFR, 6 ± 23.6; HLR, 13.7 ± 37.2 Vertical jump: BFR, 1.5 ± 7.5; HLR, 3.1 ± 12.7 |
| Laurentino | 2015 | 1RM knee extension: BFR, 85 ± 4.8, 118.8 ± 5.2; HLR, 88 ± 3.4, 119.1 ± 6 | 1RM knee extension: BFR, 38.8 ± 5; HLR, 31.1 ± 6.1 |
| Letieri | 2018 | Peak torque (leg extension): BFR, 93.7 ± 15.6, 110.4 ± 16.3; HLR, 90.6 ± 13.3, 116.3 ± 14.1 Peak torque (leg flexion): BFR, 61.3 ± 8.5, 75.4 ± 13.1; HLR, 66.5 ± 12.3, 79.2 ± 9.5 | Peak torque (leg extension): BFR, 16.7 ± 16; HLR, 25.7 ± 13.7 Peak torque (leg flexion): BFR, 14.1 ± 11.5; HLR, 12.7 ± 11.2 |
| Li | 2020 | 1RM squat: BFR, 193.7 ± 16.6, 206.5 ± 11.3; HLR, 200.5 ± 24.1, 209.2 ± 9.9 Vertical jump: BFR, 43.1 ± 4.6, 47.9 ± 5.0; HLR, 41.9 ± 2.2, 47.5±4.8 30m sprint: BFR, 4.08 ± 0.19, 3.90 ± 0.15; HLR, 4.12 ± 0.21, 4.01 ± 0.11 | 1RM squat: BFR, 12.8 ± 14.7; HLR, 8.7 ± 21 Vertical jump: BFR, 4.8 ± 4.8; HLR, 5.6 ± 4.2 30m sprint: BFR, -0.18 ± 0.17; HLR, -0.11 ± 0.18 |
| Lixandrão | 2015 | 1RM (knee extension): BFR20/40, 88.2 ± 20.9, 97.3 ± 22.4; BFR20/80, 87.1 ± 15.0, 98.6 ± 18.3；BFR40/40, 86.9 ± 19.3, 97.5 ± 20.9; BFR40/80, 89.5 ± 19.8, 100.8 ± 20.1; HLR, 86.1 ± 13.6, 104.7 ± 18.8 | 1RM (knee extension): BFR20/40, 9.1 ± 21.7, BFR20/80, 11.5 ± 16.9, BFR40/40, 10.6 ± 20.2, BFR40/80, 11.3 ± 20, HLR, 18.6 ± 16.8 |
| Luebbers | 2017 | 1RM Parallel Back Squat: BFR, 88 ± 20.6, 102.3 ± 21.7; HLR, 92.7 ± 22.5, 99.2 ± 20.7 | 1RM Parallel Back Squat: BFR, 14.3 ± 21.2; HLR, 6.5 ± 21.7 |
| Luebbers | 2014 | 1RM squat: BFR, 174.4 ± 22.2, 180.4 ± 24.9; HLR, 197 ± 35.1, 210.6 ± 38.9 | 1RM squat: BFR, 6 ± 23.7; HLR, 3.6 ± 36.7 |
| Martín-Hernández | 2012 | 1RM knee extension: BFR, 222.4 ± 32.1, 232.8 ± 30.2; HLR, 228.1 ± 33.2, 243 ± 35.7; 1RM knee flexion: BFR, 125.2 ± 22.2, 123.6 ± 18.4; HLR, 129.7 ± 21.9, 126.3 ± 20.3; | 1RM knee extension: BFR, 10.4 ± 30.2; HLR, 14.9 ± 34.5; 1RM knee flexion: BFR, -1.6 ± 20.6; HLR, -3.4 ± 21.2 |
| May | 2022 | 1RM knee extension: BFR, 73.7 ± 6.3, 86.8 ± 6.3; HLR, 70.6 ± 5.7, 83.9 ± 6.5; 1RM knee flexion: BFR, 57.9 ± 3.3, 64.0 ± 3.6; HLR, 56.2 ± 4.2, 64.2 ± 3.6; | 1RM knee extension: BFR, 13.1 ± 6.3; HLR, 13.3 ± 6.1 1RM knee flexion: BFR, 6.1 ± 3.5; HLR, 8 ± 4 |
| Mendonca | 2021 | MVC: BFR, 54.2 ± 20, 63 ± 16.6; HLR, 51.4 ± 14.7, 60.7 ± 18.5 Plantarflexion rate of torque development: BFR, 270.9 ± 112.7, 318.1 ± 84.4; HLR, 269.6 ± 127.7, 318.7 ± 120.8 | MVC: BFR, 8.8 ± 18.5; HLR, 9.3 ± 16.9 Plantarflexion rate of torque development: BFR, 47.2 ± 101.6; HLR, 49.1 ± 124.5 |
| de Lemos Muller | 2019 | 1RM knee extension: BFR, 73.5 ± 18.6, 96.2 ± 15.9; HLR, 80.6 ± 15.3, 117.5 ± 18.2 | 1RM knee extension: BFR, 22.7 ± 17.4; HLR, 36.9 ± 17 |
| Ramis | 2018 | Peak torque (knee extension): BFR, 191.1 ± 45.6, 211.2 ± 43.9; HLR, 198.2 ± 35.9, 240.9 ± 43.2 | Peak torque (knee extension): BFR, 20.1 ± 44.8; HLR, 42.7 ± 40.1 |
| Reece | 2023 | 1RM (leg extension): BFRm, 92.0 ± 33.1, 107.2 ± 32.9; HLRm, 86.5 ± 26.3, 128.9 ± 21.1; BFRf, 60.1 ± 10.2, 70.6 ± 6.9; HLRf, 64.4 ± 23.6, 82.8 ± 22.4 | 1RM (leg extension): BFRm, 15.2 ± 33.0, HLRm, 42.4 ± 24.1; BFRf, 10.5 ± 9.0, HLRf, 18.4 ± 23.0 |
| Seynnes | 2022 | 1RM (leg press): BFR, 186.4 ± 60.7, 248.9 ± 68.8; HLR, 192.0 ± 71.0, 263.9 ± 89.9 | 1RM (leg press): BFR, 62.5 ± 65.1, HLR, 71.9 ± 82.1 |
| Shao | 2023 | 1RM squat: BFR, 85.9 ± 6.1, 94.6 ± 5.4; HLR, 85.5 ± 6.5, 96.1 ± 3.9 CMJ: BFR, 282.4 ± 2.6, 291.1 ± 8.9; HLR, 281.2 ± 3.8, 287.6 ± 4.4 Horizontal jump: BFR, 276.3 ± 7.8, 284.8 ± 7.3; HLR, 280.1 ± 4.9, 286.1 ± 3.9 sprint: BFR, 3.91 ± 0.22, 3.81 ± 0.08; HLR, 3.89 ± 0.14, 3.80 ± 0.09 | 1RM squat: BFR, 8.7 ± 5.8; HLR, 10.6 ± 5.7 CMJ: BFR, 8.7 ± 7.9; HLR, 6.4 ± 4.1 Horizontal jump: BFR, 8.5 ± 7.6; HLR, 6 ± 4.5 sprint: BFR, -0.1 ± 0.19; HLR, -0.09 ± 0.13 |
| Silva | 2015 | 1RM leg extension: BFR, 35.9 ± 6.7, 40.1 ± 7.4; HLR, 27.8 ± 3.5, 37.4 ± 4.6 | 1RM leg extension: BFR, 4.2 ± 7; HLR, 9.6 ± 4.4 |
| Sousa | 2017 | Peak torque (knee extension): BFR, 70.9 ± 15.2, 86.1 ± 25.9; HLR, 66.2 ± 18.7, 94.2 ± 18.7 | Peak torque (knee extension): BFR, 15.2 ± 22.5; HLR, 28± 18.8 |
| Tang | 2022 | 1RM half squat: BFR, 111.0 ± 8.2, 119.6 ± 9.5; HLR, 107.6 ± 19.1, 112.0 ± 17.5 Peak power (sprint): BFR, 1474.2 ± 66.12, 1517.0 ± 80.8; HLR, 1434.8 ± 176.7, 1457.3 ± 170.8 | 1RM half squat: BFR, 8.6 ± 8.9; HLR, 4.4 ± 18.4 Peak power (sprint): BFR, 32.8 ± 74.6; HLR, 22.5 ± 174 |
| Thiebaud | 2013 | 1RM leg press: BFR, 99.4 ± 16.8, 107 ± 19.4; HLR, 98.7 ± 17.7, 112.2 ± 22.7 | 1RM leg press: BFR, 7.6 ± 18.2; HLR, 13.5 ± 20.7 |
| Vechin | 2015 | 1RM leg press: BFR, 280.9 ± 112.3, 324.7 ± 139.3; HLR, 181.4 ± 103.3, 271.2 ± 139.7 | 1RM leg press: BFR, 43.8 ± 128; HLR, 89.8 ± 126.1 |
| Wang | 2019 | CMJ: BFR, 51.3 ± 5.6, 54.3 ± 4; HLR, 50.8 ± 9.8, 55.6 ± 7.8 SJ: BFR, 35.0 ± 6, 41.1 ± 4.2; HLR, 34.9 ± 7.2, 42.7 ± 6.4 Peak power: BFR, 14.4 ± 1.3, 14.9 ± 2.8; HLR, 13.1 ± 2.8, 14.5 ± 1.4 30m sprint: BFR, 4.06 ± 0.22, 3.91 ± 0.14; HLR, 4.14 ± 0.20, 4.00 ± 0.13 | CMJ: BFR, 3 ± 5.1; HLR, 4.8 ± 9 SJ: BFR, 6.1 ± 5.3; HLR, 7.8 ± 6.8 Peak power: BFR, 0.5 ± 2.4; HLR, 1.4 ± 2.4 30m sprint: BFR, -0.15 ± 0.19; HLR, -0.14 ± 0.18 |
| Wang | 2022 | 1RM half squat: BFR, 180.5 ± 42.8, 198.5 ± 37.2; HLR, 196.7 ± 52.7, 230.8 ± 38.4 Horizontal jump: BFR, 262.2 ± 8.4, 263 ± 7.5; HLR, 264.7 ± 12.3, 267.8 ± 12.2 SJ: BFR, 41.9 ± 6.5, 43.3 ± 6.1; HLR, 43 ± 3.6, 45.5 ± 2.9 Peak power (SJ): BFR, 52.4 ± 6.1, 56.1 ± 2.8; HLR, 52.9 ± 2.8, 58.3 ± 3.7 CMJ: BFR, 56.9 ± 6, 57.5 ± 6.1; HLR, 59.2 ± 3.7, 61.3 ± 3.6 Peak power (CMJ): BFR, 65.9 ± 6.2, 68.5 ± 5.9; HLR, 69 ± 4.1, 72.6 ± 3.3 | 1RM half squat: BFR, 18 ± 40.3; HLR, 34.1 ± 47.2 Horizontal jump: BFR, 0.8 ± 8; HLR, 3.1 ± 12.3 SJ: BFR, 1.4 ± 6.3; HLR, 2.5 ± 3.3 Peak power (SJ): BFR, 3.7 ± 5.3; HLR, 5.4 ± 3.3 CMJ: BFR, 0.6 ± 6.1; HLR, 2.1 ± 3.7 Peak power (CMJ): BFR, 2.6 ± 6.1; HLR, 3.6 ± 3.8 |
| Wang | 2023 | 1RM back squat: BFR, 100.7 ± 12.5, 115.5 ± 10.6; HLR, 103.1 ± 7.8, 110 ± 14.2 | 1RM back squat: BFR, 14.8 ± 11.7; HLR, 6.9 ± 12.4 |
| Xie | 2023 | Peak power (bicycle): BFR, 602.2 ± 34, 640.3 ± 60.1; HLR, 600.8 ± 78, 618.2 ± 81.2 Horizontal jump: BFR, 2.27 ± 0.25, 2.45 ± 0.19; HLR, 2.17 ± 0.21, 2.27 ± 0.17 30m sprint: BFR, 5.14 ± 0.25, 4.75 ± 0.24; HLR, 4.98 ± 0.22, 4.83 ± 0.21  CMJ: BFR, 42 ± 6.3, 45.4 ± 3.6; HLR, 43 ± 6.3, 45 ± 3.7 1RM back squat: BFR, 107.5 ± 18.7, 109.7 ± 19.2; HLR, 100.3 ± 17.1, 103.8 ± 15.5 | Peak power (bicycle): BFR, 38.1 ± 52.2; HLR, 17.4 ± 79.7 Horizontal jump: BFR, 0.18 ± 0.22; HLR, 0.1 ± 0.19 30m sprint: BFR, -0.39 ± 0.25; HLR, -0.15 ± 0.21  CMJ: BFR, 3.4 ± 5.5; HLR, 2 ± 5.4 1RM back squat: BFR, 2.2 ± 18.9; HLR, 3.5 ± 16.4 |
| Yang | 2022 | SJ: BFR, 24.9 ± 5.7, 30.3 ± 7.6; HLR, 24 ± 4.3, 28.1 ± 4.4 CMJ: BFR, 28 ± 6.9, 33.5 ± 7.7; HLR, 25.4 ± 1.8, 31.1 ± 4.7  DJ: BFR, 26.9 ± 1.9, 29.7 ± 4.2; HLR, 21.9 ± 5.3, 27.1 ± 5.7 | SJ: BFR, 5.4 ± 6.9; HLR, 4.1 ± 4.4 CMJ: BFR, 5.5 ± 7.3; HLR, 5.7 ± 4.1 DJ: BFR, 2.8 ± 3.6; HLR, 5.2 ± 5.6 |
| Yasuda | 2016 | MVIC (leg extension): BFR, 91.5 ± 9.5, 104 ± 21.7; HLR, 91.9 ± 32.7, 100.9 ± 36.8 | MVIC (leg extension): BFR, 12.5 ± 18.8; HLR, 9 ± 35 |
| Zhang | 2022 | SJ: BFR, 48.3 ± 3.3, 49.1 ± 3.6; HLR, 49.5 ± 4.1, 52.1 ± 3.8 1RM back squat: BFR, 139.9 ± 10.8, 143 ± 12.6; HLR, 138.7 ± 10.7, 147.4 ± 11 | SJ: BFR, 0.8 ± 3.5; HLR, 2.6 ± 4 1RM back squat: BFR, 3.1 ± 11.8; HLR, 8.7 ± 11.4 |
