# Supplementary material for: Effect of blood-flow restricted vs heavy-load resistance training on strength, power, and speed for healthy volunteers: a systematic review and meta-analysis
Source: PeerJ. 2025 Mar 18;13:e19110. doi: 10.7717/peerj.19110 (PMC11927561; doi:10.7717/peerj.19110)
Supplement: Supplemental Information 5 [file peerj-13-19110-s005.docx]

**Funnel Plots**

**
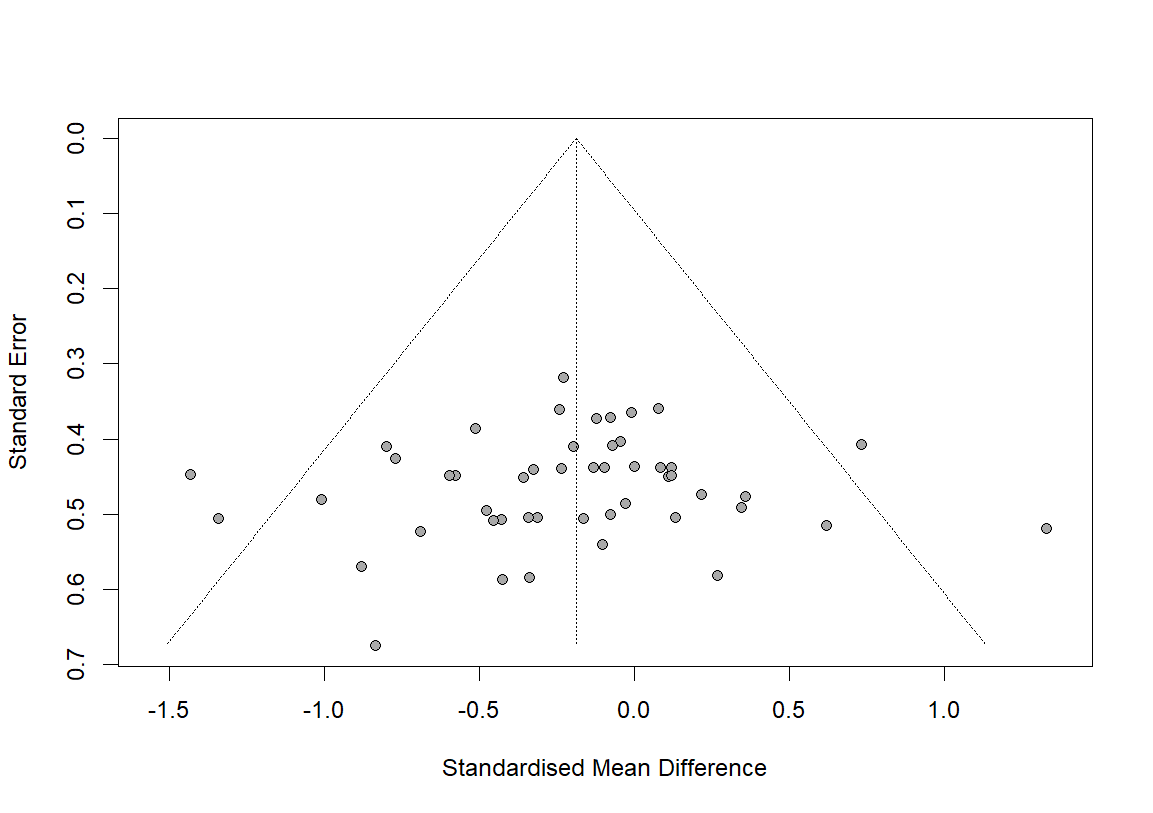
**

**Figure 1. Funnel plot for maximal strength**

**
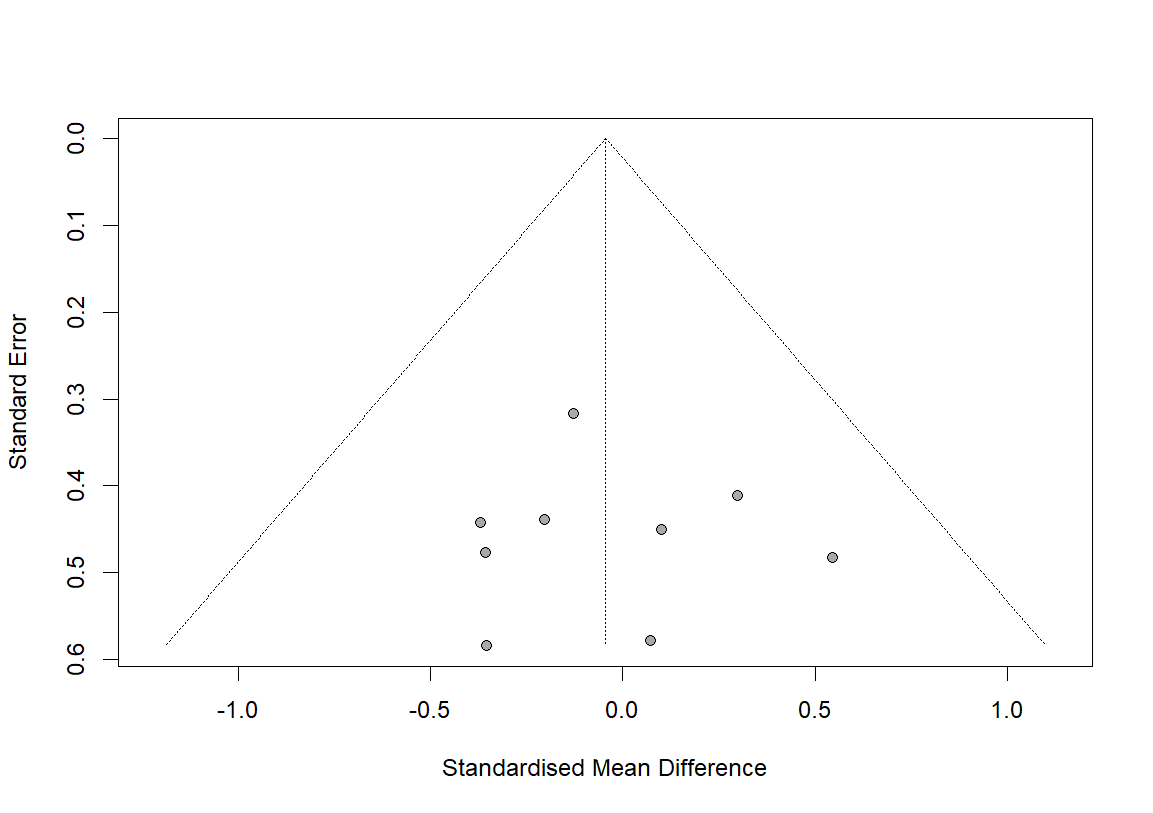
**

**Figure 2. Funnel plot for muscle power**

**
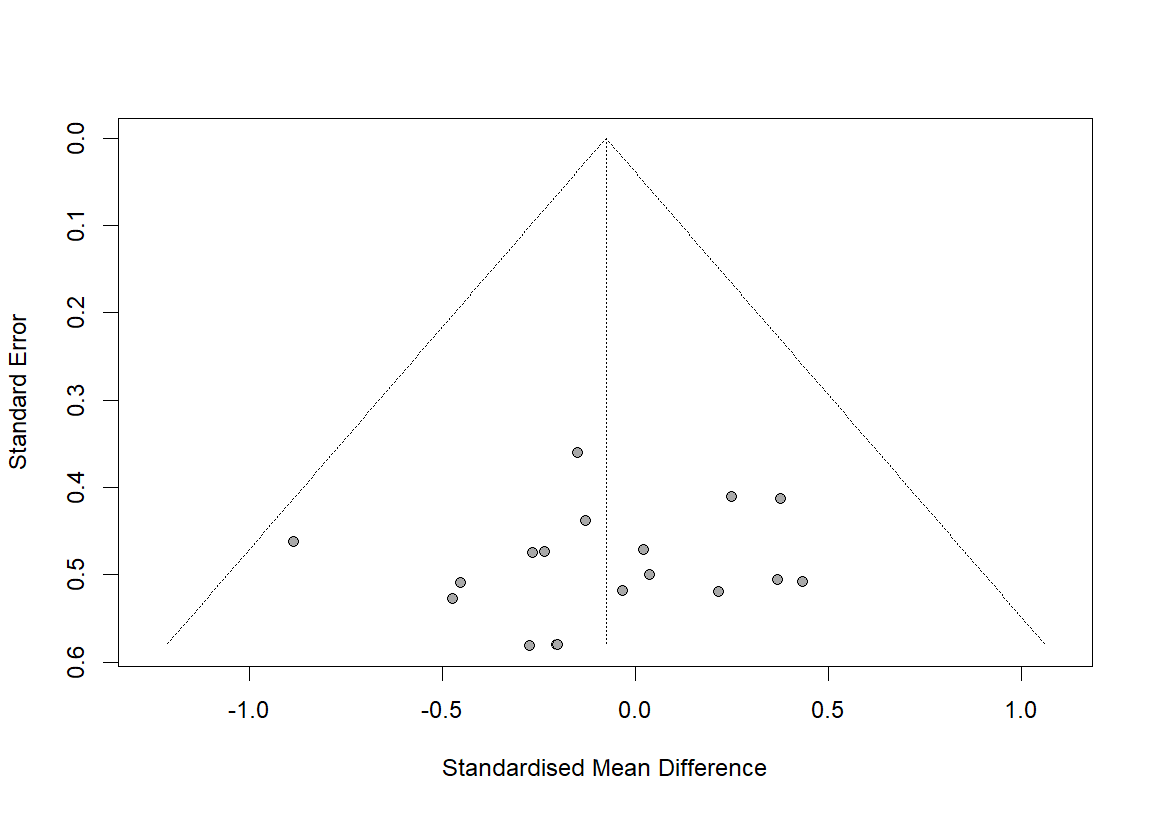
**

**Figure 3. Funnel plot for jump performance**

**
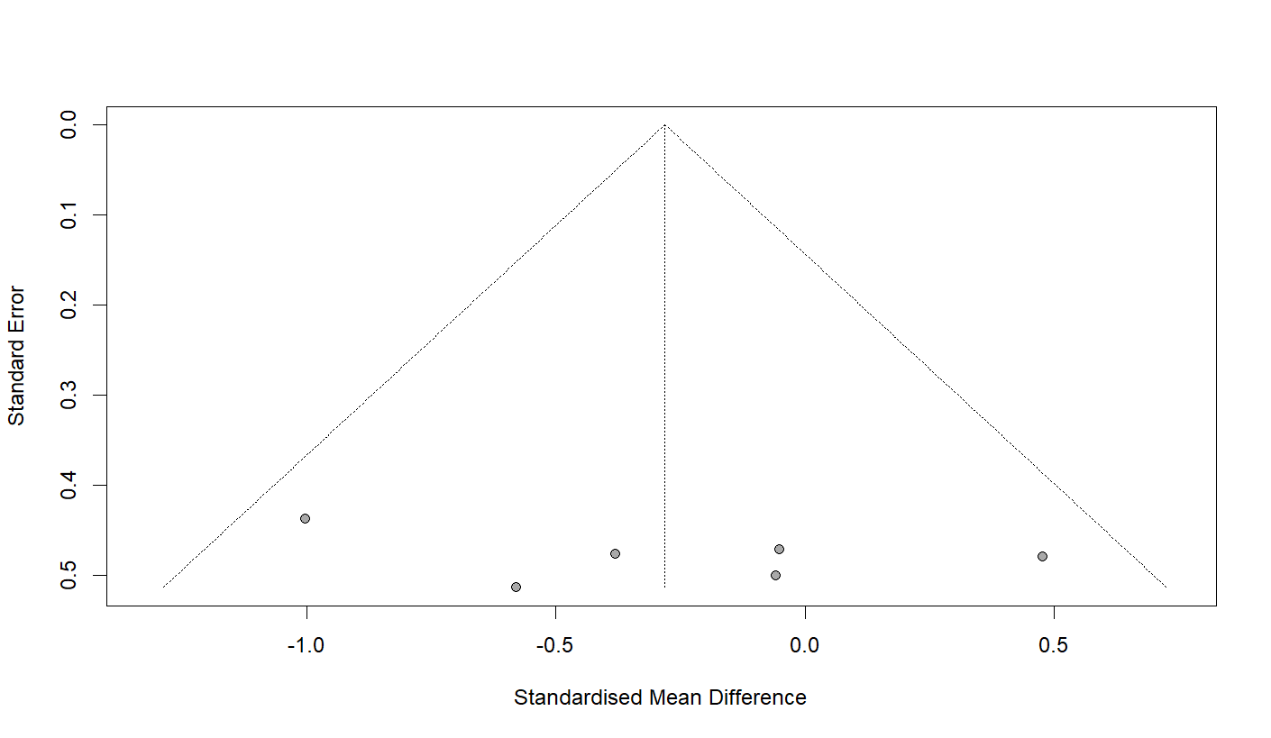
**

**Figure 4. Funnel plot for sprint performance**
